# Dynamic Complement Activation Predicts 12-Month Major Adverse Cardiovascular Events in Acute Coronary Syndromes

**DOI:** 10.3390/biomedicines14071558

**Published:** 2026-07-11

**Authors:** Florin-Leontin Lazar, Paula Anderco, Teodor Paul Kacso, Horea-Laurentiu Onea, Mihai Octavian Negrea, Diana-Raluca Lazar, Oana Stoia, Minodora Teodoru, Serban Damu, Dan-Mircea Olinic

**Affiliations:** 1 Medical Clinic Number 1, “Iuliu Haţieganu” University of Medicine and Pharmacy, 400012 Cluj-Napoca, Romania; lazar.leontin@yahoo.com (F.-L.L.); danolinic@gmail.com (D.-M.O.); 2 County Emergency Hospital, 550024 Sibiu, Romania; onea.lau@gmail.com (H.-L.O.); damu.george@gmail.com (S.D.); 3 Medical Clinical Department, Faculty of Medicine, Lucian Blaga University, 550024 Sibiu, Romania; dr.mihai.negrea@gmail.com (M.O.N.); oana.stoia@ulbsibiu.ro (O.S.); minodora.teodoru@ulbsibiu.ro (M.T.); 4 Department of Interventional Cardiology, Cluj County Emergency Hospital, 400006 Cluj-Napoca, Romania; 5 Department of Pediatric Cardiology, Pediatric Clinic No. 2, Emergency County Hospital for Children, 400394 Cluj-Napoca, Romania; lazardianaraluca@gmail.com

**Keywords:** complement, C3, C4, PCI, MACE, prognostic value

## Abstract

**Background**: The role of complement activation in acute coronary syndromes (ACS) and its prognostic significance after percutaneous coronary intervention (PCI) remain incompletely understood. **Methods**: In this prospective single-center study, 76 patients with ACS undergoing PCI were included. Serum complement components C3 and C4 were measured before PCI and again 24–48 h after the procedure. Delta values (ΔC3 and ΔC4) were calculated as post-procedural minus pre-procedural levels. The primary endpoint was major adverse cardiovascular events (MACE) at 12 months. Associations between complement parameters and outcomes were assessed using logistic regression, Cox proportional hazards models, and Kaplan–Meier analysis. A separate cohort of 19 ACS patients not undergoing PCI was analyzed as a non-PCI reference cohort. **Results**: During follow-up, 21 MACEs occurred, corresponding to an estimated MACE-free survival of 72.4%. Patients with MACE had significantly higher ΔC3 and ΔC4 values. In univariable analyses, both delta values were associated with increased MACE risk. In univariable Cox models, ΔC3 (HR 1.04, 95% CI 1.02–1.06, *p* = 0.0003) and ΔC4 (HR 1.14, 95% CI 1.05–1.23, *p* = 0.001) were associated with earlier event occurrence, and both remained significantly associated with outcomes after age-adjusted multivariable analysis (ΔC3: HR 1.03, *p* = 0.003; ΔC4: HR 1.12, *p* = 0.006). Baseline complement levels were not associated with outcomes. **Conclusions**: Periprocedural complement activation dynamics after PCI are associated with adverse outcomes in ACS and may provide incremental prognostic value.

## 1. Introduction

Acute coronary syndromes (ACS) remain a leading cause of morbidity and mortality worldwide despite major advances in revascularization and contemporary secondary prevention strategies [1]. Although percutaneous coronary intervention (PCI) has significantly improved outcomes, a substantial residual risk of major adverse cardiovascular events (MACE) persists, particularly in the early post-procedural period, despite optimal revascularization and guideline-directed therapy [2]. A MACE, typically defined as a composite of death, myocardial infarction, and repeat revascularization, is widely used as a clinically relevant endpoint in cardiovascular studies to capture overall disease burden and prognosis [3]. This residual risk highlights the need for improved risk stratification beyond traditional clinical and biochemical markers. Current risk stratification models largely rely on static biomarkers and clinical variables, potentially overlooking dynamic biological responses triggered by revascularization [4,5,6,7].

Inflammation plays a central role in the pathophysiology of ACS, contributing to plaque instability, thrombosis, and myocardial injury [8]. Conventional inflammatory markers, such as C-reactive protein, leukocyte count, and neutrophil-to-lymphocyte ratio, have been widely studied, but their ability to fully capture the complexity of the thrombo-inflammatory response remains limited [9,10]. Although emerging biomarkers such as Galectin-3 provide a more integrative assessment of both local and systemic atherosclerotic burden, they do not specifically reflect acute or procedure-related immune activation [11].

In this context, increasing attention has been directed toward the complement system, a key component of innate immunity, which is activated early in ischemic injury and has been implicated in endothelial dysfunction, leukocyte recruitment and amplification of coagulation pathways [12]. Complement activation products, including C3a and C5a, have been shown to promote leukocyte activation and endothelial dysfunction, while the terminal complement complex (C5b-9) contributes to direct cellular injury in ischemic tissues [13,14].

In the setting of PCI, additional triggers such as endothelial disruption and biomaterial interaction may further amplify complement activation beyond ischemia-driven pathways [15]. Although associations between complement activation and adverse cardiovascular outcomes are well documented, most evidence has focused on downstream activation products or single time-point measurements, with limited insight into the dynamic behavior of circulating complement components in the peri-procedural setting [13,16,17].

Complement activation in ACS can occur through three interconnected pathways [18]. The classical pathway is activated by immune complexes and oxidized lipids released during plaque rupture, while the lectin pathway is triggered by altered carbohydrate structures on ischemic endothelium and necrotic cardiomyocytes and has been implicated in experimental models of ischemia–reperfusion injury [19]. The alternative pathway acts mainly as an amplification mechanism, contributing to most of the downstream C3 and C5 activation, regardless of the initial trigger. All these pathways eventually converge at the level of C3 cleavage, leading to the generation of C3a, C5a and the terminal membrane attack complex (C5b-9) [20]. These molecules then contribute to endothelial activation, leukocyte recruitment and direct cellular injury in the reperfused myocardium [19].

Among circulating complement proteins, C3 and C4 play pivotal roles in the complement cascade, reflecting activation of the alternative and classical pathways [21]. Previous investigations have shown that circulating C3 levels are implicated in cardiometabolic risk and atherosclerotic burden, modulating the function of all major vascular cell types [22,23]. Increased levels of complement components and activation products have also been observed early after symptom onset, supporting their role in acute myocardial injury and reperfusion-related damage [24,25].

Importantly, it remains unclear whether dynamic changes in complement levels following PCI represent a nonspecific inflammatory response or reflect distinct pathophysiological processes associated with adverse outcomes. In particular, the clinical significance of dynamic changes in commonly available complement components, such as C3 and C4, has not been well defined. Therefore, the aim of the present study was to evaluate the peri-procedural dynamics of complement components C3 and C4 in patients with ACS undergoing PCI and to investigate their association with subsequent MACE. We hypothesized that post-procedural increases in complement levels reflect an amplified thrombo-inflammatory response and identify patients at higher risk of adverse cardiovascular events.

## 2. Materials and Methods

### 2.1. Study Design and Population

This was a prospective, single-center, observational study conducted at the Interventional Cardiology Department of Sibiu County Hospital between December 2024 and April 2025. A total of 76 consecutive patients were included. Patients were eligible for inclusion if they met the following criteria: age ≥ 18 years; presentation with ACS, including ST-segment elevation myocardial infarction (STEMI), non-ST-segment elevation myocardial infarction (NSTEMI), or unstable angina; and indication for coronary angiography with PCI according to current clinical guidelines. Only patients in whom serum complement components C3 and C4 could be measured at both predefined time points (pre-procedural and post-procedural) were included. Written informed consent was obtained by the responsible treating cardiologist prior to the index procedure in patients undergoing PCI or at the time of admission in patients managed without percutaneous intervention. The consent procedure was conducted in accordance with the protocol approved by the County Emergency Hospital Sibiu Clinical Research Ethics Committee (AVZ 29205, 13 November 2024). All patients were required to have complete clinical follow-up data available at 12 months, which served as the primary endpoint visit and was conducted in person. The exclusion criteria were represented by active infection or sepsis at presentation, chronic inflammatory or autoimmune disease, known hematologic or neoplastic disease under active treatment, severe hepatic insufficiency, end-stage renal disease, current treatment with immunosuppressive agents or systemic corticosteroids, recent major surgery or trauma, and refusal or inability to provide informed consent. Patients with cardiogenic shock or severe hemodynamic instability were also excluded. In addition, patients with incomplete complement measurements or unavailable follow-up data were excluded from the final analysis.

Baseline clinical, laboratory, and procedural data were collected at the time of index hospitalization. The primary endpoint assessment was performed in person at 12 months. Interim clinical status at 1, 3, and 6 months was ascertained retrospectively at the 12-month visit through review of available medical records or, when interim in-person visits had not been performed, through structured telephone interviews with patients or their treating physicians. Additionally, a subgroup of 19 patients presenting with ACS without an indication for PCI was included as a non-PCI reference cohort. In these cases, revascularization was either deferred in favor of surgical management due to anatomical suitability for coronary artery bypass grafting (CABG) or no significant obstructive coronary lesions were identified despite meeting the criteria for ACS. This subgroup was included solely to provide a baseline reference for complement levels in ACS independent of percutaneous intervention, thereby contextualizing the complement dynamics observed in the PCI cohort. Data collection for this subgroup was intentionally limited to complement measurements (C3 and C4) and basic demographic and risk factor variables; full clinical, laboratory, and procedural characterization as performed for the primary cohort was not collected, which limits formal between-group comparisons beyond complement levels.

### 2.2. Complement Measurements

Serum complement components C3 and C4 were measured at two time points: pre-procedurally, either immediately upon admission or, in STEMI cases, directly in the catheterization laboratory prior to intervention, and post-procedurally, between 24 and 48 h after the procedure. Delta values (ΔC3 and ΔC4) were defined as the difference between post- and pre-procedural levels.

### 2.3. Endpoints

The primary endpoint was major adverse cardiovascular events (MACE) at 12 months, defined as a composite of all-cause death, myocardial infarction, or repeat revascularization. Secondary endpoints included the association between complement levels and left ventricular ejection fraction (LVEF) recovery, as well as their relationship with other biomarkers, including high-sensitivity troponin, leukocyte count, and the neutrophil-to-lymphocyte ratio.

Our study protocol was carried out according to the Declaration of Helsinki and was endorsed by the County Emergency Hospital Sibiu Clinical Research Ethics Committee (AVZ 29205, on 13 November 2024).

### 2.4. Statistical Analysis

Statistical analyses were performed using R statistical software (RStudio, version 2025.05.1+513 “Mariposa Orchid” Release (ab7c1bc795c7dcff8f26215b832a3649a19fc16c, 1 June 2025)) (R Core Team, R Foundation for Statistical Computing, Vienna, Austria; available at: https://cran.r-project.org/doc/manuals/r-release/fullrefman.pdf, accessed on 5 April 2026) and RStudio (RStudio, version 2025.05.1+513 “Mariposa Orchid” Release (ab7c1bc795c7dcff8f26215b832a3649a19fc16c, 1 June 2025)). Continuous variables are presented as mean ± standard deviation or median (interquartile range), as appropriate. Categorical variables are expressed as counts and percentages.

Normality was assessed using the Shapiro–Wilk test. Between-group comparisons were performed using the Mann–Whitney U test for non-normally distributed variables.

Associations with outcomes were evaluated using univariable logistic regression and Cox proportional hazards models. Results are reported as odds ratios (OR) or hazard ratios (HR) with 95% confidence intervals (CI).

Kaplan–Meier survival analysis was used to estimate event-free survival, with results presented as cumulative survival rates. Spearman correlation analysis was used to assess relationships between complement levels and inflammatory biomarkers. A two-sided *p*-value < 0.05 was considered statistically significant. Spearman’s rank correlation coefficient (rather than Pearson’s) was selected given the non-normal distribution of several biomarkers, as determined by the Shapiro–Wilk testing described above; no additional correlation analyses beyond those originally reported were performed.

## 3. Results

### 3.1. Baseline Characteristics

A total of 76 patients were included, of whom 59 (77.6%) were male. Cardiovascular risk factors were highly prevalent, with hypertension observed in 58 patients (76.3%), diabetes mellitus in 30 (39.5%), active smoking in 25 (32.9%), and obesity in 17 (22.4%). A history of cardiovascular disease was noted in a high proportion of patients, including prior myocardial infarction in 14 (18.4%) and previous PCI in 10 (13.2%). Multivessel coronary artery disease was present in 45 patients (62.5%). Baseline characteristics are summarized in Table 1.

Of the 75 patients with complete ACS-subtype data, 18 (24.0%) presented with unstable angina, 40 (53.3%) with NSTEMI, and 17 (22.7%) with STEMI; one patient with missing subtype data was excluded from this comparison. The 12-month MACE rate differed significantly across subtypes (UA 11.1%, NSTEMI 40.0%, STEMI 17.6%; χ^2^ *p* = 0.043), with NSTEMI carrying the highest event rate in this cohort.

### 3.2. Procedural Characteristics

Most patients were treated with drug-eluting stents (84.2%), while drug-coated balloons were used in 5.3% and a hybrid strategy in 10.5% of cases. The majority of lesions were de novo, with in-stent restenosis observed only rarely (1.3%), and ostial involvement present in 10.5% of lesions. Bifurcation lesions accounted for 17.1% of cases, while angiographically significant calcification was identified in 27.5%.

Lesion preparation was predominantly performed using semi-compliant balloons (62.7%), followed by non-compliant balloons (26.7%) and cutting balloons (9.3%). Intravascular lithotripsy was used in a small proportion of cases (1.3%), while no procedures required rotational atherectomy or specialty high-pressure balloons. Among patients treated with drug-eluting stents, the mean stent diameter was 3.21 mm (IQR 2.84–3.50), with a total stented length of 30.9 mm (IQR 18–40). Table 2 summarizes the main procedural characteristics.

### 3.3. Complement Dynamics and Clinical Outcomes

Mean C3 levels increased from 133.8 ± 31.1 to 136.0 ± 30.2, with a median ΔC3 of 3.0 (IQR 0.0–7.0). Similarly, C4 increased from 26.9 ± 7.6 to 29.2 ± 8.6, with a median ΔC4 of 2.0 (IQR 0.7–3.15).

Despite modest average increases, both ΔC3 and ΔC4 demonstrated substantial inter-individual variability (ΔC3: −63.0 to 75.0; ΔC4: −12.3 to 20.0).

**Figure 1:**
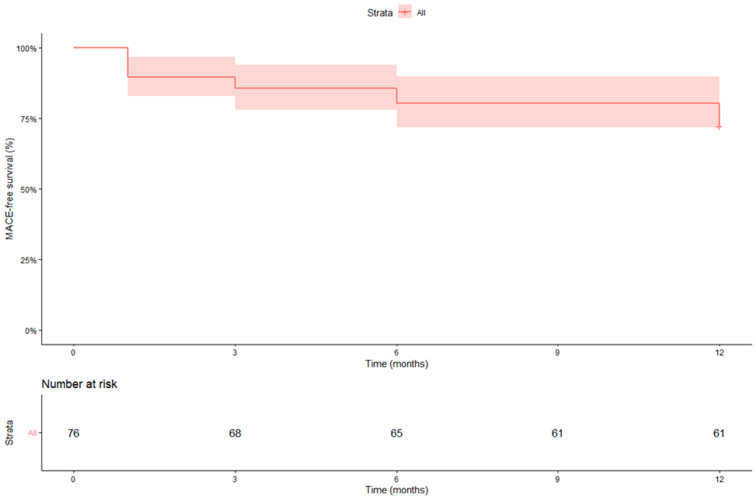
Kaplan–Meier Curve for 12-Month MACE-Free Survival.

At 12 months, a total of 21 MACEs occurred. Kaplan–Meier analysis showed an estimated MACE-free survival of 72.4% (95% CI 63.0–83.2). Most events occurred early after the procedure, with a steeper decline in the survival curve within the first months, followed by a more gradual decrease thereafter (Figure 1).

### 3.4. Complement and Clinical Outcomes

Patients experiencing MACE exhibited significantly greater increases in complement levels compared with those without events (ΔC3: 7 [2–24] vs. 2 [0–4.5], *p* = 0.013; ΔC4: 4 [0.6–7] vs. 1.1 [0.9–2.2], *p* = 0.014). Baseline complement levels were not associated with outcomes.

In univariable logistic regression, both ΔC3 and ΔC4 were associated with 12-month MACE. Each unit increase in ΔC3 was associated with 7% higher odds of MACE (OR 1.07, 95% CI 1.02–1.13, *p* = 0.008), while ΔC4 was associated with a 21% increase (OR 1.21, 95% CI 1.06–1.41, *p* = 0.010).

In Cox proportional hazards analysis, ΔC3 and ΔC4 were both significantly associated with time to MACE. Each unit increase in ΔC3 was associated with a higher hazard of earlier MACE occurrence (HR 1.04, 95% CI 1.02–1.06, *p* = 0.0003), and ΔC4 with a similar effect (HR 1.14, 95% CI 1.05–1.23, *p* = 0.001). These findings were consistent with results from univariable logistic regression and non-parametric comparisons.

**Figure 2:**
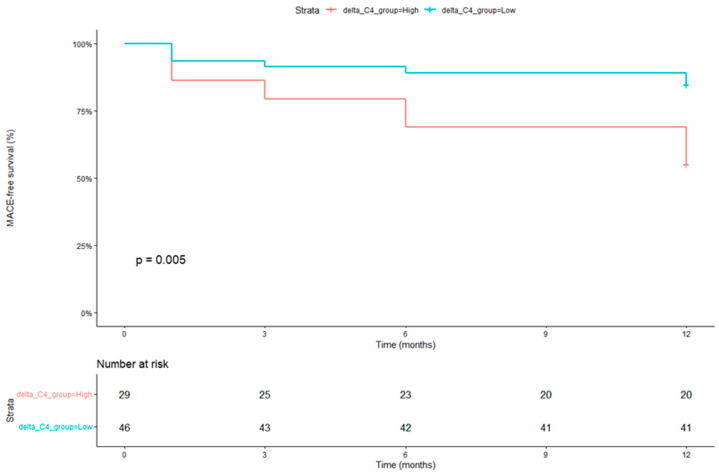
Time-to-Event Analysis of 12-Month MACE According to ΔC3 Levels.

**Figure 3:**
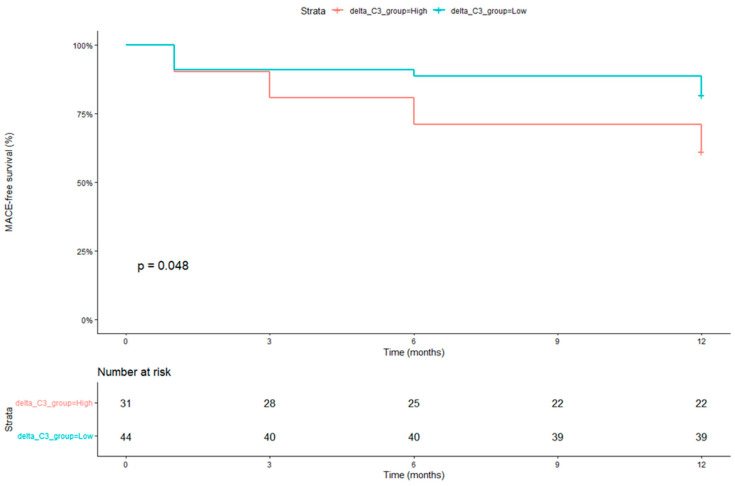
Time-to-Event Analysis of 12-Month MACE According to ΔC4 Levels.

To further illustrate the relationship between complement dynamics and clinical outcomes, patients were stratified according to the median values of ΔC3 and ΔC4, and Kaplan–Meier survival analyses were performed (Figure 2 and Figure 3), demonstrating significantly lower MACE-free survival in patients with ΔC3 or ΔC4 values above the median compared with those at or below the median.

In multivariable Cox analysis adjusted for age, both ΔC3 (HR 1.03, 95% CI 1.01–1.05, *p* = 0.003) and ΔC4 (HR 1.12, 95% CI 1.03–1.21, *p* = 0.006) remained associated with earlier MACE occurrence. Baseline complement levels and age were not associated with outcomes.

To address discrimination, Harrell’s concordance statistic (C-index) was computed for each Cox model: 0.668 (SE 0.070) for the univariable ΔC3 model, 0.664 for the univariable ΔC4 model, 0.683 for the age-adjusted ΔC3 model, and 0.689 for the age-adjusted ΔC4 model (in addition to the concordance of 0.644 reported below for the age + ΔCRP model). These values indicate modest discrimination, consistent with the exploratory, hypothesis-generating framing of these analyses in a small cohort.

To assess whether the prognostic association of complement dynamics was independent of conventional inflammatory marker kinetics, a separate Cox proportional hazards model incorporating age and ΔCRP (the change in C-reactive protein between post- and pre-procedural measurements) was constructed, restricted to two predictors in keeping with the events-per-variable constraints of this cohort (19 events). In this model (*n* = 71, 19 events), ΔCRP remained significantly associated with time to MACE (HR 1.01, 95% CI 1.00–1.02, *p* = 0.03), while age showed a borderline, non-significant association (HR 1.04, 95% CI 1.00–1.09, *p* = 0.08); model concordance was 0.644. The proportional-hazards assumption for ΔCRP showed a borderline violation on Schoenfeld residual testing (*p* = 0.019), a finding not uncommon in small cohorts, and this model should therefore be interpreted as exploratory.

### 3.5. Correlation with Inflammatory Biomarkers

**Figure 4:**
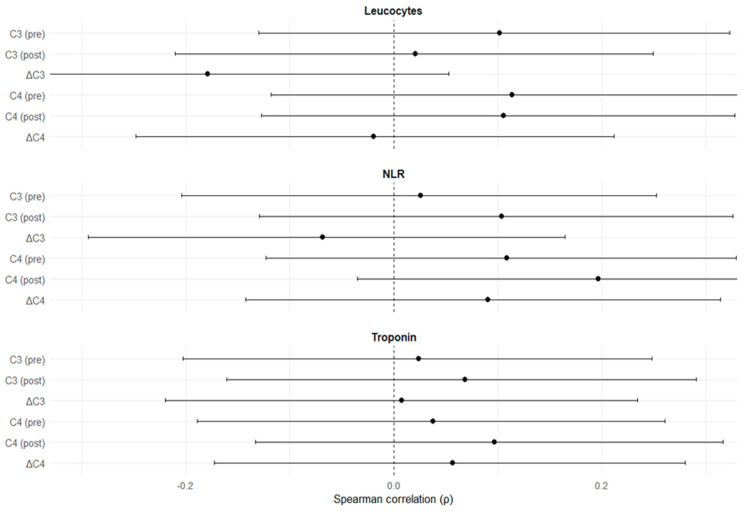
Correlation between Complement Components and Biomarkers.

No significant correlations were observed between complement components (C3, C4, or their delta values) and conventional inflammatory or myocardial injury biomarkers, including high-sensitivity troponin, leukocyte count, and neutrophil-to-lymphocyte ratio (all |ρ| < 0.2, *p* > 0.05), as illustrated in Figure 4.

No association was found between complement changes and CRP dynamics. However, post-procedural C4 levels showed a modest correlation with CRP (ρ = 0.29, *p* = 0.015), while C3 demonstrated only a non-significant trend.

### 3.6. Exploratory Analyses and Non-PCI Reference Cohort

No statistically significant differences in complement levels were observed according to multivessel disease status. However, patients with multivessel disease showed a numerically greater increase in ΔC3 compared with those with single-vessel disease, without reaching statistical significance (*p* = 0.075). No significant correlation was observed between complement changes and LVEF. The main baseline characteristics of the non-PCI reference cohort are summarized in Table 3.

Baseline complement levels in the non-PCI reference cohort were 120.8 ± 14.1 for C3 and 28.8 ± 8.2 for C4. When compared with the PCI-treated population, mean C3 levels were higher in patients undergoing intervention (133.8 ± 31.1 vs. 120.8 ± 14.1); however, this difference did not reach statistical significance (*p* ≈ 0.06) and, in the absence of serial post-procedural complement sampling in the non-PCI cohort, cannot be interpreted as evidence of a procedure-specific effect as opposed to the natural course of the ACS event itself. In contrast, C4 levels were comparable between groups (26.9 ± 7.6 vs. 28.8 ± 8.2, *p* ≈ 0.30).

## 4. Discussion

The complement system represents a central component of innate immunity and has been increasingly recognized as a key mediator in the pathophysiology of atherosclerosis and ACS. Complement activation contributes to endothelial dysfunction, promotes leukocyte recruitment, and amplifies inflammatory signaling within the atherosclerotic plaque [26]. Growing evidence suggests that elevated C3 levels, a key component of the complement system, are associated with an increased risk of cardiovascular disease in humans [22]. In addition, early elevations in C3, C4, and C5b-9 observed within 24 h of admission in patients with ACS further support the involvement of complement activation in the pathophysiological processes underlying myocardial damage [24].

Building on this background, our study demonstrates that dynamic changes in complement components, rather than baseline levels, are associated with clinical outcomes in patients presenting with ACS. Specifically, increases in C3 and C4 levels following percutaneous coronary intervention were associated with the occurrence and timing of major adverse cardiovascular events at 12 months and remained associated with outcomes after parsimonious multivariable adjustment. These findings suggest that complement activation reflects an active and evolving inflammatory response that may contribute to adverse cardiovascular outcomes beyond the initial ischemic insult.

While logistic regression was used to assess the association between complement parameters and the occurrence of MACE, Cox proportional hazards analysis was employed to account for the time-to-event nature of the data. Notably, complement dynamics were significantly associated not only with the occurrence but also with the timing of MACE, suggesting that heightened complement activation may identify patients at risk for earlier adverse events. This reinforces the value of time-to-event analyses in capturing the prognostic impact of inflammatory pathways in ACS.

To further explore the clinical relevance of complement dynamics, patients were stratified according to the median values of ΔC3 and ΔC4, allowing comparison between groups with relatively higher versus lower complement activation. This approach provided an intuitive and clinically interpretable visualization of risk, particularly in the context of Kaplan–Meier analyses. Although dichotomization facilitates interpretation, it may result in loss of information compared with analyses based on continuous variables. Accordingly, these findings were interpreted alongside the primary continuous analyses to ensure a more robust assessment of prognostic significance.

The observed variability in ΔC3 and ΔC4 in our cohort suggests a heterogeneous inflammatory response among patients, potentially reflecting differences in plaque characteristics, procedural complexity, or individual immune reactivity. Importantly, the lack of significant correlation between complement changes and conventional inflammatory markers such as C-reactive protein, leukocyte count, or neutrophil-to-lymphocyte ratio indicates that complement activation may represent a distinct biological pathway not fully captured by traditional biomarkers.

Although 12-month MACE rates differed significantly by ACS subtype in this cohort (highest in NSTEMI), the magnitude of the periprocedural rise in ΔC3 and ΔC4 did not differ significantly across UA, NSTEMI, and STEMI (Kruskal–Wallis *p* = 0.62 and *p* = 0.52, respectively), suggesting that complement dynamics are not simply a proxy for ACS subtype itself.

The stronger association observed for ΔC4 compared with ΔC3 may have biological relevance. C4 is cleaved through the classical and lectin pathways and is not directly involved in the alternative pathway. Therefore, an increase in C4 may more specifically reflect activation of these initiating pathways. By contrast, C3 represents the central convergence point of all three complement pathways and is also continuously involved in the alternative pathway amplification loop. This may make ΔC3 a broader but less pathway-specific marker of complement turnover. In this context, the stronger association between ΔC4 and adverse outcomes may suggest that classical and lectin pathway activation, potentially related to plaque rupture, endothelial injury, and ischemia–reperfusion, contributes to post-procedural complement-mediated injury. However, this interpretation remains exploratory and should be confirmed using pathway-specific activation products in larger cohorts [27,28].

The absence of a significant correlation between complement dynamics and CRP may be explained by differences in biological kinetics and compartmentalization. CRP is a hepatic acute-phase reactant, largely driven by IL-6 signaling, and typically reflects a delayed systemic inflammatory response. In contrast, complement proteins are constitutively present in the circulation and can be activated rapidly and locally at sites of endothelial injury, plaque rupture, thrombosis, and ischemia–reperfusion. Moreover, many downstream effects of complement activation occur within the vascular wall and reperfused myocardium, through anaphylatoxin-mediated leukocyte recruitment, endothelial activation, and membrane attack complex formation. These local processes may not be directly captured by circulating CRP levels. Therefore, the lack of correlation between CRP and complement dynamics supports the interpretation that ΔC3 and ΔC4 may reflect a biological pathway distinct from conventional systemic inflammatory markers [9,29,30].

In this context, our comparison with a non-PCI reference cohort provides additional insight. Baseline complement levels were broadly comparable between groups, suggesting that complement activation is already present at the time of ACS presentation, independent of revascularization strategy. The trend toward higher baseline C3 levels in the PCI group may reflect a greater inflammatory burden or more advanced coronary disease in patients selected for intervention. It may also suggest that procedural vascular injury and reperfusion may further amplify complement activation, as suggested in previous publications [31]. This dual contribution, disease-related and procedure-related, may explain the strong association between complement dynamics and adverse outcomes [32]. In contrast, the lack of difference in C4 levels is consistent with the concept that complement pathways may be differentially activated and regulated in the setting of ACS [33,34].

From a clinical perspective, these findings raise the hypothesis that periprocedural complement dynamics may provide incremental prognostic information in ACS. While the exploratory nature of this work precludes definitive conclusions, the consistent associations observed across multiple analytical approaches support the biological plausibility of this concept. Prospective validation in larger, multicenter cohorts is required to establish the clinical utility of complement monitoring. In this context, prior trials of complement-targeted therapy are informative. Pexelizumab, an anti-C5 agent, was investigated as adjunctive therapy in STEMI, although its effects on infarct size and clinical outcomes were inconsistent across trials [35]. Therefore, the present findings should not be interpreted as supporting routine complement-directed therapy in ACS, but rather as supporting further investigation of complement activation as a mechanistic and prognostic pathway [35,36].

Beyond complement, several other biomarker classes are being investigated for risk stratification in ACS. Hematologic and metabolic indices derived from routine blood counts, such as the Systemic Inflammatory Response Index and the triglyceride-glucose-body mass index, have recently been reported to independently predict MACE after PCI in ACS cohorts [37,38], and galectin-3, previously studied by our group in the context of systemic atherosclerosis [11], continues to be explored as a complementary fibrosis-related marker. These indices reflect partially overlapping but mechanistically distinct processes and could, in principle, be combined with complement dynamics in future multimarker models; such integration was beyond the scope of the present hypothesis-generating analysis.

Several limitations should be acknowledged. This was a single-center study with a relatively small sample size, which may limit the generalizability of the findings and the robustness of multivariable analyses. The observational design precludes conclusions regarding causality. The multivariable Cox model was intentionally parsimonious, adjusted for age and baseline complement levels only, yielding an approximate events-per-variable ratio of 7:1. However, residual confounding cannot be excluded. For the same reason, we deliberately did not expand the multivariable models to include additional candidate confounders (e.g., ACS subtype, renal function, diabetes, multivessel disease, lesion complexity, or procedural complications); with only 21 MACEs, doing so would have introduced a comparable or greater risk of model overfitting and unstable estimates. Accordingly, the findings should be interpreted as hypothesis-generating and exploratory, requiring confirmation in larger prospective cohorts. Additionally, complement activation was assessed using circulating C3 and C4 levels, which do not fully capture the complexity of the complement cascade, including activation fragments such as C3a, C5a, or the membrane attack complex. In this sense, C3 and C4 should be regarded as surrogates of complement consumption and activation rather than as direct activation products [26]; dedicated assays for C3a, C5a, Bb, C4d, or sC5b-9 will be required to confirm pathway-specific activation in future studies. Beyond the concordance statistics reported in Section 3.4, a formal comparison with established risk scores (e.g., net reclassification or integrated discrimination improvement relative to the GRACE score) could not be performed, as the variables required to compute the GRACE score (e.g., systolic blood pressure, heart rate, and Killip class at presentation) were not systematically collected in this cohort. Finally, the non-PCI reference cohort was characterized only with respect to complement levels and basic demographic variables; the absence of full clinical, laboratory, and procedural data for this subgroup limits the depth of between-group comparisons. Rather than being viewed solely as a constraint, this relatively small, single-center cohort should be regarded as a starting point: future multicenter studies enrolling larger and more diverse ACS populations will be needed both to validate the present associations and to support more complex multivariable models incorporating additional clinical, laboratory, and inflammatory covariates, such as ΔCRP.

## 5. Conclusions

In patients with ACS, dynamic increases in complement components C3 and C4 following percutaneous coronary intervention were associated with 12-month major adverse cardiovascular events, whereas baseline levels were not predictive. These findings suggest that complement activation may reflect an evolving inflammatory response with potential prognostic significance beyond traditional biomarkers. The comparison with a non-PCI ACS reference cohort suggests that complement activation may already be present at baseline in ACS, while peri-procedural complement changes after PCI may provide additional prognostic information. Given the modest sample size and single-center design, these results require prospective validation in larger cohorts before complement dynamics can be considered for clinical risk stratification. Future studies should also explore the mechanistic contribution of specific complement pathways and the potential role of complement inhibition as a therapeutic strategy in high-risk ACS patients.

## Figures and Tables

**Table 1:** Baseline characteristics stratified by 12-month MACE status.

Parameter	No MACE (*n* = 55)	MACE (*n* = 21)	*p*-Value	Test
**Demographics**				
Male sex, *n* (%)	43 (78.2)	16 (76.2)	1.000	Fisher’s exact
Age, years	63.64 ± 11.97	70.33 ± 11.93	0.035	*t*-test
ACS presentation				
Unstable angina, *n* (%)	16 (29.6)	2 (9.5)	0.043	Chi-square (overall, 3 groups)
NSTEMI, *n* (%)	24 (44.4)	16 (76.2)	0.043	Chi-square (overall, 3 groups)
STEMI, *n* (%)	14 (25.9)	3 (14.3)	0.043	Chi-square (overall, 3 groups)
**Cardiovascular risk factors**				
Hypertension, *n* (%)	42 (76.4)	16 (76.2)	1.000	Fisher’s exact
Diabetes mellitus, *n* (%)	20 (37.0)	9 (42.9)	0.792	Fisher’s exact
Current smoking, *n* (%)	21 (38.2)	4 (19.0)	0.172	Fisher’s exact
Obesity, *n* (%)	11 (20.0)	6 (28.6)	0.539	Fisher’s exact
**Medical history**				
Previous myocardial infarction, *n* (%)	10 (18.2)	4 (19.0)	1.000	Fisher’s exact
Previous PCI, *n* (%)	7 (12.7)	3 (14.3)	1.000	Fisher’s exact
Previous CABG, *n* (%)	3 (5.5)	1 (4.8)	1.000	Fisher’s exact
Previous stroke, *n* (%)	7 (12.7)	2 (9.5)	1.000	Fisher’s exact
Chronic heart failure, *n* (%)	2 (3.6)	4 (19.0)	0.046	Fisher’s exact
Atrial fibrillation, *n* (%)	9 (16.4)	6 (28.6)	0.333	Fisher’s exact
**Laboratory parameters**				
CRP (pre-procedural), mg/L	4.80 (1.40–11.70)	11.70 (5.10–81.30)	0.014	Mann–Whitney U
CRP (post-procedural), mg/L	7.80 (3.90–12.10)	51.10 (7.30–112.90)	0.002	Mann–Whitney U
Leukocyte count, ×10^9^/L	10.30 (8.22–12.48)	9.48 (7.95–12.01)	0.853	Mann–Whitney U
Neutrophil-to-lymphocyte ratio	2.68 (1.79–4.55)	4.25 (2.50–7.20)	0.108	Mann–Whitney U
Hemoglobin, g/dL	13.98 ± 2.00	13.50 ± 2.49	0.439	*t*-test
LDL cholesterol, mg/dL	117.20 (74.70–153.90)	95.45 (79.00–144.75)	0.585	Mann–Whitney U
HDL cholesterol, mg/dL	36.05 ± 9.14	31.92 ± 9.25	0.173	*t*-test
Triglycerides, mg/dL	164.00 (109.75–202.75)	111.00 (94.00–187.00)	0.397	Mann–Whitney U
Creatinine, mg/dL	0.93 (0.81–1.08)	0.97 (0.81–1.22)	0.848	Mann–Whitney U
High-sensitivity troponin, ng/L	340.00 (4.00–1371.00)	1095.00 (226.00–3788.00)	0.015	Mann–Whitney U
BNP, pg/mL	179.00 (35.93–487.00)	376.00 (155.75–619.25)	0.126	Mann–Whitney U
LVEF, %	50.00 (45.00–55.00)	40.00 (27.50–50.00)	0.004	Mann–Whitney U

Continuous variables are presented as mean ± SD (Welch’s *t*-test) when normally distributed, or median (IQR) (Mann–Whitney U test) when non-normally distributed, per Shapiro–Wilk testing. Categorical variables are presented as *n* (%) and compared using Fisher’s exact test.

**Table 2:** Procedural characteristics stratified by 12-month MACE status.

Parameter	No MACE (*n* = 55)	MACE (*n* = 21)	*p*-Value	Test
**Lesion type**				
In-stent restenosis, *n* (%)	1 (3.2)	0 (0.0)	1.000	Fisher’s exact
**Treatment strategy**				
Drug-eluting stent, *n* (%)	46 (83.6)	18 (85.7)	1.000	Fisher’s exact
Drug-coated balloon, *n* (%)	4 (7.3)	0 (0.0)	0.571	Fisher’s exact
Hybrid strategy, *n* (%)	2 (3.7)	1 (4.8)	1.000	Fisher’s exact
**Lesion preparation**				
Semi-compliant balloon, *n* (%)	34 (63.0)	13 (61.9)	1.000	Fisher’s exact
Non-compliant balloon, *n* (%)	14 (25.9)	6 (28.6)	1.000	Fisher’s exact
Cutting balloon, *n* (%)	4 (7.4)	3 (14.3)	0.392	Fisher’s exact
Intravascular lithotripsy, *n* (%)	0 (0.0)	1 (4.8)	0.280	Fisher’s exact
Rotablation, *n* (%)	0 (0.0)	0 (0.0)	1.000	Fisher’s exact
Super-high-pressure balloon, *n* (%)	0 (0.0)	0 (0.0)	1.000	Fisher’s exact
**Lesion characteristics**				
Bifurcation lesion, *n* (%)	6 (10.9)	7 (33.3)	0.037	Fisher’s exact
Ostial involvement, *n* (%)	4 (7.3)	4 (19.0)	0.206	Fisher’s exact
Calcified lesion, *n* (%)	11 (22.0)	10 (47.6)	0.046	Fisher’s exact
Multivessel disease, *n* (%)	31 (59.6)	14 (70.0)	0.588	Fisher’s exact
Vessel diameter, mm	3.50 (2.88–3.50)	3.00 (2.75–3.50)	0.372	Mann–Whitney U
Lesion length, mm	28.00 (18.00–42.00)	32.00 (19.50–47.00)	0.576	Mann–Whitney U
Total stent length, mm	26.00 (18.00–38.00)	34.00 (18.00–45.00)	0.365	Mann–Whitney U
Mean stent diameter, mm	3.15 (2.75–3.50)	3.25 (2.94–3.50)	0.893	Mann–Whitney U

Continuous variables are presented as mean ± SD (Welch’s *t*-test) when normally distributed, or median (IQR) (Mann–Whitney U test) when non-normally distributed, per Shapiro–Wilk testing. Categorical variables are presented as *n* (%) and compared using Fisher’s exact test.

**Table 3:** Baseline characteristics of the non-PCI reference cohort.

Overall Population (*n* = 19)	
Age, mean ± SD	64.4 ± 12.7
Male sex (%)	63.2
Hypertension (%)	31.6
Diabetes mellitus (%)	31.6
Smoking (%)	47.4
Obesity (%)	10.5
C3, mean ± SD	120.8 ± 14.1
C4, mean ± SD	28.8 ± 8.2

## Data Availability

The original contributions presented in this study are included in this article. Further inquiries can be directed to the corresponding authors.

## References

[1] Roth G.A., Mensah G.A., Johnson C.O., Addolorato G., Ammirati E., Baddour L.M., Barengo N.C., Beaton A.Z., Benjamin E.J., Benziger C.P. (2020). Global Burden of Cardiovascular Diseases and Risk Factors, 1990–2019. J. Am. Coll. Cardiol..

[2] Byrne R.A., Rossello X., Coughlan J.J., Barbato E., Berry C., Chieffo A., Claeys M.J., Dan G.-A., Dweck M.R., Galbraith M. (2023). 2023 ESC Guidelines for the management of acute coronary syndromes. Eur. Heart J..

[3] Bosco E., Hsueh L., McConeghy K.W., Gravenstein S., Saade E. (2021). Major adverse cardiovascular event definitions used in observational analysis of administrative databases: A systematic review. BMC Med. Res. Methodol..

[4] Galli M., Abbate A., Bonaca M.P., Crea F., Forte M., Frati G., Gaudino M., Gibson C.M., Gorog D.A., Mehran R. (2026). Residual cardiovascular risk in coronary artery disease: From pathophysiology to established and novel therapies. Nat. Rev. Cardiol..

[5] Dhindsa D.S., Sandesara P.B., Shapiro M.D., Wong N.D. (2020). The Evolving Understanding and Approach to Residual Cardiovascular Risk Management. Front. Cardiovasc. Med..

[6] Giubilato S., Lucà F., Abrignani M.G., Gatto L., Rao C.M., Ingianni N., Amico F., Rossini R., Caretta G., Cornara S. (2023). Management of Residual Risk in Chronic Coronary Syndromes. Clinical Pathways for a Quality-Based Secondary Prevention. J. Clin. Med..

[7] Patel K.V., Pandey A., De Lemos J.A. (2018). Conceptual Framework for Addressing Residual Atherosclerotic Cardiovascular Disease Risk in the Era of Precision Medicine. Circulation.

[8] Hansson G.K. (2005). Inflammation, Atherosclerosis, and Coronary Artery Disease. N. Engl. J. Med..

[9] Ridker P.M. (2005). C-reactive protein, inflammation, and cardiovascular disease: Clinical update. Tex. Heart Inst. J..

[10] Intravaia R.C.M., Tognola C., Maloberti A., Brucato F., Campione E., Giannattasio C., Zuin M., Mojoli M., Abrignani M.G., Oliva F. (2026). The role of inflammation in acute coronary syndrome: A systematic review on biochemical inflammatory assessment and anti-inflammatory therapies. Nutr. Metab. Cardiovasc. Dis..

[11] Onea H.L., Homorodean C., Lazar F.L., Negrea M.O., Calin T., Bitea I.C., Teodoru M., Nechita V.I., Olteanu A.L., Olinic D.-M. (2025). Galectin-3 Reflects Systemic Atherosclerosis in Patients with Coronary Artery Disease. Medicina.

[12] Ricklin D., Lambris J.D. (2013). Complement in immune and inflammatory disorders: Pathophysiological mechanisms. J. Immunol..

[13] Vahldieck C., Löning S., Hamacher C., Fels B., Rudzewski B., Nickel L., Weil J., Nording H., Baron L., Kleingarn M. (2024). Dysregulated complement activation during acute myocardial infarction leads to endothelial glycocalyx degradation and endothelial dysfunction via the C5a:C5a-Receptor1 axis. Front. Immunol..

[14] Hausenloy D.J., Yellon D.M. (2013). Myocardial ischemia-reperfusion injury: A neglected therapeutic target. J. Clin. Investig..

[15] Ekdahl K.N., Huang S., Nilsson B., Teramura Y. (2016). Complement inhibition in biomaterial- and biosurface-induced thromboinflammation. Semin. Immunol..

[16] Van Greevenbroek M.M., Arts I.C., Van Der Kallen C.J., Geijselaers S.L., Feskens E.J., Jansen E.H., Schalkwijk C.G., Stehouwer C.D., Hertle E. (2014). Distinct associations of complement C3a and its precursor C3 with atherosclerosis and cardiovascular disease: The CODAM study. Thromb. Haemost..

[17] Hertle E., Van Greevenbroek M.M.J., Arts I.C.W., Van Der Kallen C.J.H., Feskens E.J.M., Schalkwijk C.G., Stehouwer C. (2014). Complement activation products C5a and sC5b-9 are associated with low-grade inflammation and endothelial dysfunction, but not with atherosclerosis in a cross-sectional analysis: The CODAM study. Int. J. Cardiol..

[18] Dunkelberger J.R., Song W.C. (2010). Complement and its role in innate and adaptive immune responses. Cell Res..

[19] Walsh M.C., Bourcier T., Takahashi K., Shi L., Busche M.N., Rother R.P., Solomon S.D., Ezekowitz R.A.B., Stahl G.L. (2005). Mannose-Binding Lectin Is a Regulator of Inflammation That Accompanies Myocardial Ischemia and Reperfusion Injury. J. Immunol..

[20] Wu G., Hu W., Shahsafaei A., Song W., Dobarro M., Sukhova G.K., Bronson R.R., Shi G.-P., Rother R.P., Halperin J.A. (2009). Complement regulator CD59 protects against atherosclerosis by restricting the formation of complement membrane attack complex. Circ. Res..

[21] Li X.X., Woodruff T.M. (2025). The complement system: Biology, pathology, and therapeutic interventions. Pharmacol. Rev..

[22] Garcia-Arguinzonis M., Diaz-Riera E., Peña E., Escate R., Juan-Babot O., Mata P., Badimon L., Padro T. (2021). Alternative C3 Complement System: Lipids and Atherosclerosis. Int. J. Mol. Sci..

[23] Li Y., Zeng H., Zhong X. (2025). Complement C3 in panvascular disease: A central integrator of immune signaling and vascular remodeling. Clin. Sci..

[24] Bavia L., Lidani K.C.F., Andrade F.A., Sobrinho M.I.A.H., Nisihara R.M., De Messias-Reason I.J. (2018). Complement activation in acute myocardial infarction: An early marker of inflammation and tissue injury?. Immunol. Lett..

[25] Kluge K.E., Halvorsen S., Andersen G.Ø., Hansen C.H., Seljeflot I., Tønnessen T., Lunde I.G., Helseth R. (2025). Combined complement and coagulation activation in ST-elevation myocardial infarction: Associations with myocardial injury and dysfunction. Front. Immunol..

[26] Hok K.D., Rich H.E., Shadid A., Gunamalai L., Weng-Mills T., Thandavarayan R.A., Banda N.K., Doursout M.-F., Restrepo M.I., Shivshankar P. (2025). Functional Roles of the Complement Immune System in Cardiac Inflammation and Hypertrophy. Int. J. Mol. Sci..

[27] Hurler L., Toonen E.J.M., Kajdácsi E., van Bree B., Brandwijk R.J.M.G.E., de Bruin W., Lyons P.A., Bergamaschi L. (2022). Distinction of early complement classical and lectin pathway activation via quantification of C1s/C1-INH and MASP-1/C1-INH complexes using novel ELISAs. Front. Immunol..

[28] Horváth Z., Csuka D., Vargova K., Kovács A., Leé S., Varga L., Préda I., Zsámboki E.T., Prohászka Z., Kiss R.G. (2016). Alternative complement pathway activation during invasive coronary procedures in acute myocardial infarction and stable angina pectoris. Clin. Chim. Acta.

[29] Liuzzo G., Biasucci L.M., Gallimore J.R., Grillo R.L., Rebuzzi A.G., Pepys M.B., Maseri A. (1994). The Prognostic Value of C-Reactive Protein and Serum Amyloid A Protein in Severe Unstable Angina. N. Engl. J. Med..

[30] Pepys M.B., Hirschfield G.M. (2003). C-reactive protein: A critical update. J. Clin. Investig..

[31] Tucker B., Vaidya K., Cochran B.J., Patel S. (2021). Inflammation during Percutaneous Coronary Intervention—Prognostic Value, Mechanisms and Therapeutic Targets. Cells.

[32] Kluge K.E., Langseth M.S., Andersen G.Ø., Halvorsen S., Opstad T.B., Arnesen H., Tønnessen T., Seljeflot I., Helseth R. (2022). Complement activation in association with clinical outcomes in ST-elevation myocardial infarction. Am. Heart J. Plus Cardiol. Res. Pract..

[33] Merle N.S., Church S.E., Fremeaux-Bacchi V., Roumenina L.T. (2015). Complement System Part I “ Molecular Mechanisms of Activation and Regulation. Front. Immunol..

[34] Fujita T. (2002). Evolution of the lectin–complement pathway and its role in innate immunity. Nat. Rev. Immunol..

[35] Granger C.B., Mahaffey K.W., Weaver W.D., Theroux P., Hochman J.S., Filloon T.G., Rollins S., Todaro T.G., Nicolau J.C., Ruzyllo W. (2003). Pexelizumab, an Anti-C5 Complement Antibody, as Adjunctive Therapy to Primary Percutaneous Coronary Intervention in Acute Myocardial Infarction: The COMplement inhibition in Myocardial infarction treated with Angioplasty (COMMA) Trial. Circulation.

[36] Armstrong P., Granger C., Adams P., Hamm C. (2007). Pexelizumab for Acute ST-Elevation Myocardial Infarction in Patients Undergoing Primary Percutaneous Coronary Intervention: A Randomized Controlled Trial. JAMA.

[37] Alhawamdeh N., Bani Hani D.A., Alshraideh J.A., Yousef A.S., Saleh A. (2026). Novel inflammatory markers predict cardiovascular events after percutaneous coronary intervention in patients with acute coronary syndrome. Heart Lung.

[38] Becirovic E., Becirovic M., Ljuca K., Becirovic A., Babic M., Ljuca N., Jusic Z.B., Begagic E., Mujakovic E., Terzic A. (2025). Integration of Hematologic and Metabolic Biomarkers for Outcome Prediction in Acute Coronary Syndromes Without ST Elevation. Cureus.

